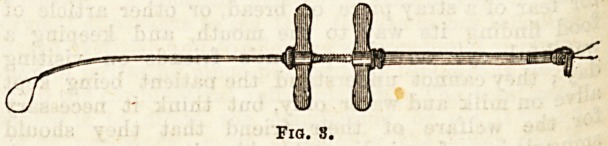# Operative Treatment of Mucous Polypi of the Nose

**Published:** 1893-02-18

**Authors:** 


### ROYAL INFIRMARY, EDINBURGH.

### Operative Treatment of Mucous Polypi of the Nose.

As patients suffering from mucous polypi of the nose seldom seek surgical aid until the growths have attained such a size as to cause a considerable amount of inconvenience, it is unnecessary to consider here the non-operative methods of treatment by astringent or caustic applications, which are only appropriate to tumours of comparatively small dimensions.

The *symptoms* mo t complained of are blocking of the nasal passages, interfering with respiration and the sense of smell; alteration in the tone of the voice; and a watery discharge from the nose, all of which are exaggerated in damp weather.

From the point of view of *treatment*, it is important to bear in mind that these tumours grow from the superior spongy bone, never from the septum floor or outer side of the nasal passage. They are almost always pedunculated, the point of junction with the normal mucous membrane being the thinnest, narrowest, and weakest part. They are usually multiple, but vary much in size, some being so small as to be impossible of detection, and so escape removal along with their larger fellows. Their subsequent growth is often a source of disappointment, and the surgeon should never fail to warn his patient of the possibility of its occurrence.

The majority of polypi can be examined and attacked from the anterior nares. They appear as pyriform semi-gelatinous, greyish masses, soft and freely movable. A smaller number can only be seen with the aid of the rhinoscope from the posterior nares.

Let us first consider the treatment of those that present *anteriorly*. Pain is obviated by the use of a 20 per cent. solution of cocaine, painted or sprayed over the mucous membrane. Dr. McBride prefers a mixture of cocaine and menthol, which he finds more efficacious.

The choice of instruments is practically between (1) the polypus forceps, (2) the cold snare, and (3) the electro-cautery snare.

In this hospital there is some divergence of opinion as to which is the best, the general surgeons following the teaching and practice of Syme in using the long, strong, slightly curved forceps, serrated throughout their whole length (Fig. 1). These are gently introduced, grasping the tumour as near as possible to the base, and by steady, careful twisting and pulling detaching the growth from the mucous membrane.

In the special clinic Dr. McBride seldom uses the forceps except in cases where it is found impossible to pass the loop of a snare round the polypus. His objections to this instrument being its less degree of

Fig. 1.

certainty except with bright illumination, and the greater amount of pain inflicted.

Of the two snares he prefers the cold one, because by it he is able, with very little pain to the patient, to rapidly and completely tear cut the whole growth. Where the electro-cautery snare is used there is no bleeding, but this slight advantage, Dr. McBride thinks, is more than counter-balanced by the pain inflicted in slowly severing the polypus, and by the fact that part of the growth is left behind, the wire not passing through the pedicle close to its attachment to the normal mucous membrane. In this way the chance of recurrence is much increased. With both varieties of snare piano-wire should be employed.

Fig. 2.

A most important step in the removal of mucous polypi is the complete destruction, by cauterisation or otherwise, of the root or pedicle. This may be done by scraping with the finger-nail or a sharp spoon, or, better still, by the application of some fused chromic acid on the end of a probe. Dr. McBride neutralises any excess of chromic acid by the application of some bicarbonate of soda. Spraying the part with rectified spirit was long ago suggested by Mr. A. G. Miller, of Edinburgh, and gives excellent results. When the cautery is used to remove the tumour the pedicle may well be destroyed by a second application.

Fig. 3.

Those polypi which project posteriorly and lie on the palate are usually single, and with the aid of the posterior-rhinoscope (fig. 2), may be seized with a Jarvis snare (fig. 3) and removed entire.

## Figures and Tables

**Fig. 1. f1:**
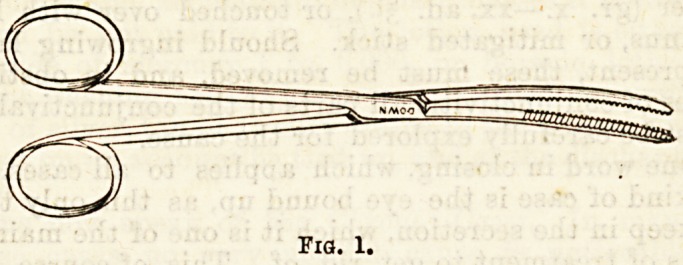


**Fig. 2. f2:**
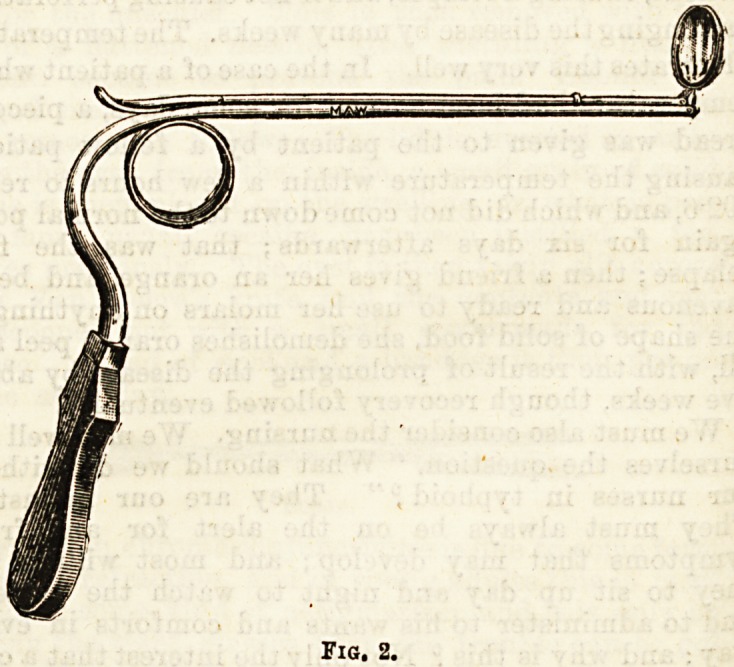


**Fig. 3. f3:**